# The Hippocratic Oath

**Published:** 1881-12

**Authors:** 


### THE HIPPOCRATIC OATH.

The most curious medical monument of antiquity is the famous Hippocratic oath, the faithful observance of which secured good success in life, and general esteem. The oath is as follows:

"I swear, by Apollo, the physician, by Æsculapius, by Hygeia and Panacea, and all the gods and goddesses calling them to witness that I will fulfil religiously, according to the best of my power and judgment, the solemn promise and the written bond which I now do make. I will honor, as my parents, the master who has taught me this art, and endeavor to minister to all his necessities. I will consider his children as my own brothers, and will teach them my profession, should they express a desire to follow it, without remuneration or written bond. I will admit to my lessons, my discourses, and all my other methods of teaching, my own sons, and those of my tutor, and those who have been inscribed as pupils and have taken the medical oath; but no one else. I will prescribe such a course of regimen as may be best suited to the condition of my patients; according to the best of my power and judgment, seek to preserve them from anything that might prove injurious. No inducement shall ever lead me to administer poison, nor will I ever be the author of such advice: neither will I contribute to an abortion. I will maintain religiously the purity and integrity, both of my conduct and my art. I will not cut any one for the stone, but will leave that operation to those who cultivate it. Into whatever dwellings I may go, I will enter them with the sole view of succoring the sick, abstaining from all injurious views and corruption, especially from any immodest action towards women and men, freeman or slaves. If during my attendance, or even unprofessionally, in common life, I happen to see or hear of any circumstances which should not be revealed, I will consider them a profound secret, and observe on the subject a religious silence. May I, if I rigidly observe this, my oath, and do not break it, enjoy good success in life and in (the practice of) my art, and obtain general esteem forever. Should I transgress and become a perjurer, may remorse be my lot."—*Cincinnati Lancet and Clinic.*

